# Single-Cell RNA Sequencing Reveals the Migration of Osteoclasts in Giant Cell Tumor of Bone

**DOI:** 10.3389/fonc.2021.715552

**Published:** 2021-08-24

**Authors:** Wenyu Feng, Mingwei He, Xiaohong Jiang, Huijiang Liu, Tianyu Xie, Zhaojie Qin, Qian Huang, Shijie Liao, Chengsen Lin, Juliang He, Jiake Xu, Jie Ma, Yun Liu, Qingjun Wei

**Affiliations:** ^1^Department of Trauma Orthopedic and Hand Surgery, First Affiliated Hospital of Guangxi Medical University, Nanning, China; ^2^Guangxi Collaborative Innovation Center for Biomedicine, Guangxi Medical University, Nanning, China; ^3^Department of Orthopedic, Affiliated Minzu Hospital of Guangxi Medical University, Nanning, China; ^4^Department of Orthopedics, The First People’s Hospital of Nanning, Nanning, China; ^5^Department of Spinal Bone Disease, First Affiliated Hospital of Guangxi Medical University, Nanning, China; ^6^Department of Bone and Soft Tissue Surgery, Guangxi Medical University Cancer Hospital, Nanning, China; ^7^School of Biomedical Sciences, The University of Western Australia, Perth, WA, Australia; ^8^Department of Medical Oncology, First Affiliated Hospital of Guangxi Medical University, Nanning, China

**Keywords:** single cell RNA sequence, giant cell tumor of bone (GCTB), osteolysis, osteoclast, migration

## Abstract

Giant cell tumor of bone (GCTB) is benign tumor that can cause significant osteolysis and bone destruction at the epiphysis of long bones. Osteoclasts are thought to be highly associated with osteolysis in GCTB. However, the migration of osteoclasts in GCTB remains unclear. A deeper understanding of the complex tumor microenvironment is required in order to delineate the migration of osteoclasts in GCTB. In this study, samples were isolated from one patient diagnosed with GCTB. Single-cell RNA sequencing (scRNA-seq) was used to detect the heterogeneity of GCTB. Multiplex immunofluorescence staining was used to evaluate the cell subtypes identified by scRNA-seq. A total of 8,033 cells were obtained from one patient diagnosed with GCTB, which were divided into eight major cell types as depicted by a single-cell transcriptional map. The osteoclasts were divided into three subsets, and their differentiation trajectory and migration status were further analyzed. Osteoclast migration may be regulated *via* a series of genes associated with cell migration. Furthermore, four signaling pathways (RANKL, PARs, CD137 and SMEA3 signaling pathway) were found to be highly associated with osteoclast migration. This comprehensive single-cell transcriptome analysis of GCTB identified a series of genes associated with cell migration as well as four major signaling pathways that were highly related to the migration of osteoclasts in GCTB. Our findings broaden the understanding of GCTB bionetworks and provides a theoretical basis for anti-osteolysis therapy against GCTB in the future.

## Introduction

Giant cell tumor of bone (GCTB) is destructive osteolytic benign tumor that often affect the epiphysis of long bones and can lead to severe motor dysfunction (1). GCTB usually occurs in young adults between the ages of 30 and 40 years (2). The main pathologic feature of GCTB is severe osteolysis that is thought to be caused by osteoclasts, which are multinucleated cells derived from the monocyte/macrophage lineage (3). Surgical resection is the main treatment for GCTB. However, repetitive operations after the primary surgery due to local recurrence can lead to serious functional complications (4). The use of drugs designed to inhibit the function of osteoclasts, such as denosumab or zoledronic acid, can contribute to symptomatic relief but do not inhibit the development of GCTB (5). An in-depth understanding of the mechanism behind osteoclast-mediated osteolysis by GCTB is therefore of critical importance.

The combination of receptor activator of nuclear factor-κ B (RANK) and its ligand (RANKL) regulates osteoclast activity, which involves osteoclast attachment to the bone surface and digestion of the bone matrix *via* the secretion of acid (H^+^ and Cl^-^) and proteinases (cathepsin K) (6). These phenomena are not only observed under normal physiologic conditions but also in the setting of pathologic bone resorption, such as by bone tumors such as GCTB (3). However, why osteoclasts migrate directly to the lesion and why osteoclast-mediated osteolysis occurs remains unknown. The answer may be related to the tumor microenvironment, in which cells directly communicate with each other through cellular contact and interaction with the extracellular matrix (7). Analysis of the unique types of GCTB tumor microenvironments can reveal key factors involved in osteoclast migration and osteoclast-mediated osteolysis that may serve as future targets of novel therapeutic strategies. We hypothesized that osteoclast migration is influenced by the complex and dynamic features of the GCTB tumor microenvironment.

Given that conventional bulk RNA-sequencing (RNA-seq) is based on the hypothesis that every gene is expressed equally by every cell, it is therefore unable to accurately characterize the heterogeneity of tumor microenvironments at the cell-type level (8). With advancement in the scRNA-seq technique, heterogeneous tissues can be delineated at the single-cell level (9). This technology permits the massively parallel characterization of thousands of cells at the transcriptome level and can better explain cell–cell interactions. However, relatively few studies have been published on the single-cell transcriptome of GCTB. Here we used scRNA-seq to investigate the intratumoral heterogeneity of GCTB. A relative comprehensive single-cell transcriptome profiling of GCTB was performed, and gene features and cellular dynamics associated with osteoclast migration were identified.

## Materials and Methods

### Patient and Sample Collection

This work was approved by the First Affiliated Hospital of Guangxi Medical University (No.2019KY-E-153) and complied with all relevant ethical regulations. The patient with GCTB whose clinical and cellular data was used in this study provided informed consent. The patient’s basic information, X-ray and MRI imaging, and pathology slides are shown in **Supplementary Figure 1** and **Supplementary Table 1**. Fresh specimens acquired at the time of surgical resection were collected in cold Hank’s balanced salt solution (cat. no. 311-512-CL; Wisent Bio Products) containing 1% antibiotic-antimycotic (cat. no. 15240062; Thermo Fisher) and transported to the laboratory as soon as possible.

### Preparation of the Single-Cell Suspension

Fresh tumor specimens were mechanically isolated and enzymatically digested to produce single-cell suspensions. The tumor was washed two times with Dulbecco’s phosphate-buffered saline (DPBS; cat. no. 14190250; Gibco) and minced on ice. The tumor was then subjected to enzymatic type II (cat. no. 17101015; Thermo Fisher) digestion in a water bath at 37 ° to generate GCTB cell suspensions. A 100-μm nylon cell strainer (cat. no. 352340; Falcon) was used to filter impurities. Red blood cells (RBC) were lysed with RBC lysis buffer (cat. no. 1966634; Invitrogen) containing DNase I (1 unit/ml) and removed *via* centrifugation. The cell suspensions were refiltered with a 40 μm nylon cell strainer (cat. no. 352340; Falcon) to capture the isolated cells. The dissociated cells were stained with trypan blue (0.4%; cat. no. 420301; Thermo Fisher) to calculate cell viability and diluted with DPBS containing 1% fetal bovine serum (FBS; cat. no. 10091148; Thermo Fisher) into appropriate concentrations for the next step.

### Preparation of Single-Cell Suspensions for Library Construction and scRNA-Sequencing

Single-cell suspensions of GCTB were uploaded into an emulsion droplet using 10× Genomics Chromium Controller (version 3) to generate single-cell gel bead-in-emulsions (GEMs). mRNA in drops was subjected to reverse-transcription reactions, and cDNA amplification was performed according to the manufacturer’s instructions. The 10× libraries were sequenced with the HiSeq Xten (Illumina, San Diego, CA) sequencing platform.

### Pre-Processing of scRNA-Sequencing Data

CellRanger (version 4.0.0) was used to convert the preliminary sequencing results (bcl files) generated from HiSeq Xten into fastq files. The fastq files were then aligned to the human genome reference sequence GRCh38. A Seurat package (version 3.1.1) in R software (version 3.6.3, R-Foundation, Vienna, Austria) was used to generate a gene-barcode matrix containing the barcoded cells and gene expression counts. Low-quality cells (gene numbers < 200 or > 4,000 and with a percentage of mitochondrial genes of > 10%) were directly filtered. A total of 8,033 cells were ultimately included for further bioinformatic analysis.

### Cell Clustering Analysis, Visualization, and Annotation

Cell-clustering and sub-clustering analyses were performed with the FindClusters function of the Seurat package (resolution = 0.1). Identified cell clusters and sub-clusters were presented with uniform manifold approximation and projection (UMAP) analysis. The cell clusters were annotated based on highly expressed genes and some canonical cellular markers from the literature.

### Pseudotime Trajectory Analysis

The evolutionary processes of osteoclasts and mononuclear macrophages were analyzed using the Monocle 3 package (version 0.2.3.0; https://coletrapnelllab.github.io/monocle3/). Cells were organized into discontinuous trajectories. The identified genes varied in their expression over these trajectories. In the selection of cell sequencing parameters, the starting point of the cell was selected based on the results of RNA velocity analysis. Six genes (CD74, HLA-DRA, CD14, ACP5, CTSK, and ATP6V0D2) that play an important role in the maturation of osteoclasts were also subjected to pseudotime trajectory analysis. The evolutionary process of CD8^+^T cells was analyzed using the Monocle 2 package (version 2.14.0; http://cole-trapnell-lab.github.io/monocle-release/) (10). The following parameters were set: Mean expression >0.1, num_cells_expressed >=10. GZMK and GZMA also underwent pseudotime trajectory analysis.

### Cell–Cell Communication Analysis

CellPhoneDB analysis, which is based on the interaction between ligands and receptors (11, 12), was performed using a CellPhoneDB Python package (version 0.22; https://github.com/Teichlab/cellphonedb). We also identified some relevant cell type-specific interactions based on ligand–receptor pairs (P value < 0.05).

### CellChat Analysis

CellChat, an intercellular interaction analysis tool that studies ligand receptor action in specific signaling pathways (13), was performed using the CellChat R package (version 0.0.1; https://github.com/sqjin/CellChat). A CellChat object was created using the R package process. Cell information was added into the meta slot of the object. The ligand–receptor interaction database was set, and the matching receptor inference calculation was performed. The graphic visualization parameter was nPatterns = 5.

### Functional Enrichment Analysis

Gene set variation analysis (GSVA) was used to identify the gene set that was significantly enriched in each subset of mononuclear macrophages (14). We downloaded all gene sets from the Molecular Signatures Database MSigDB (https://www.gseamsigdb.org/gsea/downloads.jsp).

### Single-Cell Regulatory Network Inference and Clustering (SCENIC) Analysis

SCENIC is a suitable method for using transcription factors (TFs) to guide the discovery of cellular states from scRNA-seq data (15). The R package for SCENIC (version 1.2.4) was used as previously described to perform a transcription factor network inference analysis (16). Briefly, a read-count matrix from the Seruat S4 object was inputted. The matrix was filtered using default parameters and used to establish a gene regulatory network. Differentially activated TFs in different cell types were identified using the Wilcoxon rank sum test. TFs with an adjusted P-value <0.05 and logFC >0.1 were considered significant.

### RNA Velocity Analysis

RNA velocity predicts dynamic changes in transcription and the future state of individual cells (17). We obtained.loom files through velocyto.py (version 0.17.17). The.loom files were uploaded to the velocyto.R package (version 0.6.0). The following parameters were set: fit.quantile = 0.02; kCells = 25; and DeltaT = 1. Arrows indicating velocity vector were projected onto the UMAP plot.

### Cellular Spatial Organization Mapper (CSOmap) Analysis

CSOmap (version 1.0) is an analytical method for calculating the spatial information of cells by using the interactions between ligand receptors (18). We generated an affinity matrix between cells by integrating thousands of ligand-receptor pairs. The resulting high-dimensional affinity matrix is embedded in three-dimensional space. The contribution of ligand receptor genes to spatial information was then calculated. Finally, genes highly expressed by C2_Mature osteoclasts were selected for computer overexpression and knockdown to observe changes in spatial structure.

### Multiplex Immunohistochemistry (IHC) Staining

Multiplex immunohistochemistry staining was performed according to the manufacturer’s instructions using the PANO 7-plex IHC kit (Panovue, Beijing, China) (19, 20). Sections with a thickness of 3 μm were incubated overnight at 4°C with primary antibodies: anti-ACP5 (cat. no. 11594-1-AP; Rabbit; 1:300; Proteintech), anti-ATP6V0D2 (cat. no. bs-12548R; Rabbit; 1:300; Bioss), and anti-CKLF (cat. no. ab250213; 1:300; Rabbit; Abcam). Secondary antibodies were then used to incubate the sections at room temperature for 15 min, after which the tyramide signal amplification (TSA) plus working solution was applied. Other primary antibodies were applied to the slides, and the steps mentioned above were repeated until the last antibody was used. Finally, 4-6-diamidino-2-phenylindole (DAPI; SigmaAldrich) was used to stain the nuclei and multispectral images were collected with a confocal laser-scanning microscope (LSM880; Zeiss).

### Statistical Analysis

All statistical analysis and figures were generated using R software (version 3.6.3). A p-value < 0.05 was considered statistically significant.

## Results

### GCTB Cellular Contribution

**Figure 1:**
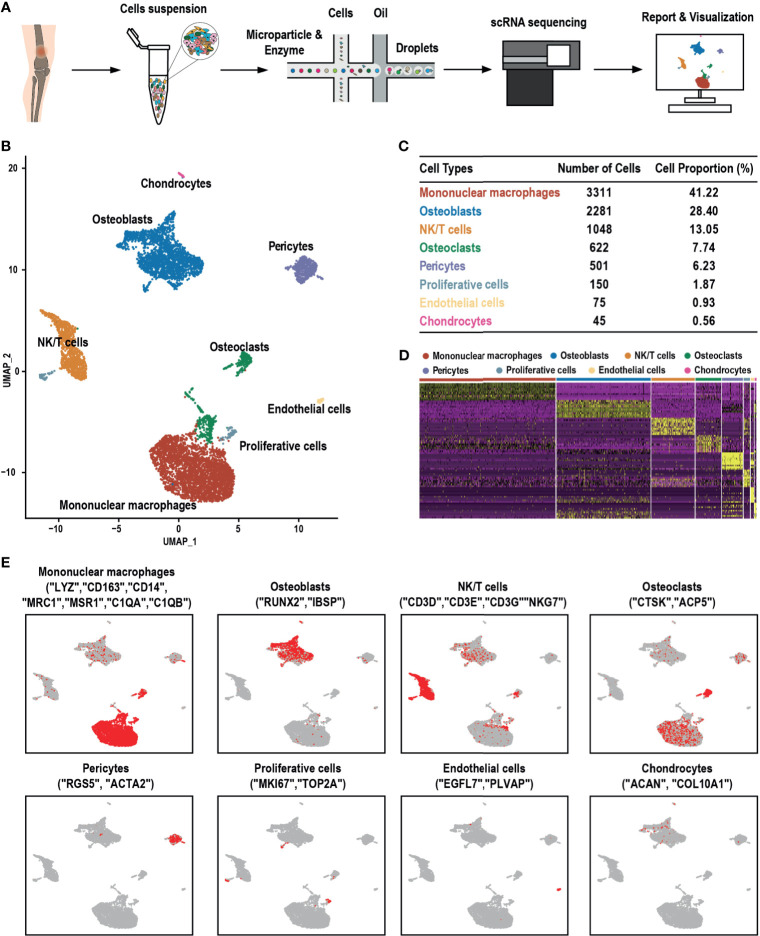
scRNA-seq clustering analysis of GCTB. **(A)** Workflow depicting the collection and processing of GCTB tumor specimens for scRNA-seq. **(B)** UMAP plot of 8,033 cells demonstrating the eight main cell types in GCTB. **(C)** Cell number and percentage of the assigned cell types. **(D)** The heat-map shows and highlights the differentially expressed genes of each cluster. **(E)** UMAP plots show the expression of representative well-known markers across cell types in GCTB. scRNA-seq, single-cell RNA sequencing; GCTB, giant cell tumor of bone; UMAP, uniform manifold approximation and projection.

scRNA-seq analysis was performed on samples obtained from a patient diagnosed with GCTB during tumor resection (**Figure 1A**). Following initial quality control assessment, the single-cell transcriptomic data of 8,033 cells from the primary GCTB lesion were used for further analysis. Eight main cell clusters were identified (**Figure 1B**): mononuclear macrophages, osteoblasts, NK/T cells, osteoclasts, pericytes, proliferating cells, endothelial cells, and chondrocytes (**Figure 1C**). The canonical markers of each cell cluster were as follows **(** **Figure 1D** **)**: [1] LYZ, CD163, CD14, MRC1, MSR1, C1QA, and C1QB for mononuclear macrophages (20); [2] RUNX2 and IBSP for osteoblasts (21, 22); [3] CD3D, CD3E, CD3G, and NKG7 for NK/T cells (23–25); [4] CTSK and ACP5 for osteoclasts (26); [5] RGS5 and ACTA2 for pericytes (24); [6] MKI67 and TOP2A for proliferative cells (24, 27); [7] EGFL7 and PLVAP for endothelial cells (28, 29); and [8] ACAN and COL10A1 for chondrocytes (24, 30) **(** **Figure 1E** **)**.

### Heterogeneity of Mononuclear Macrophages

**Figure 2:**
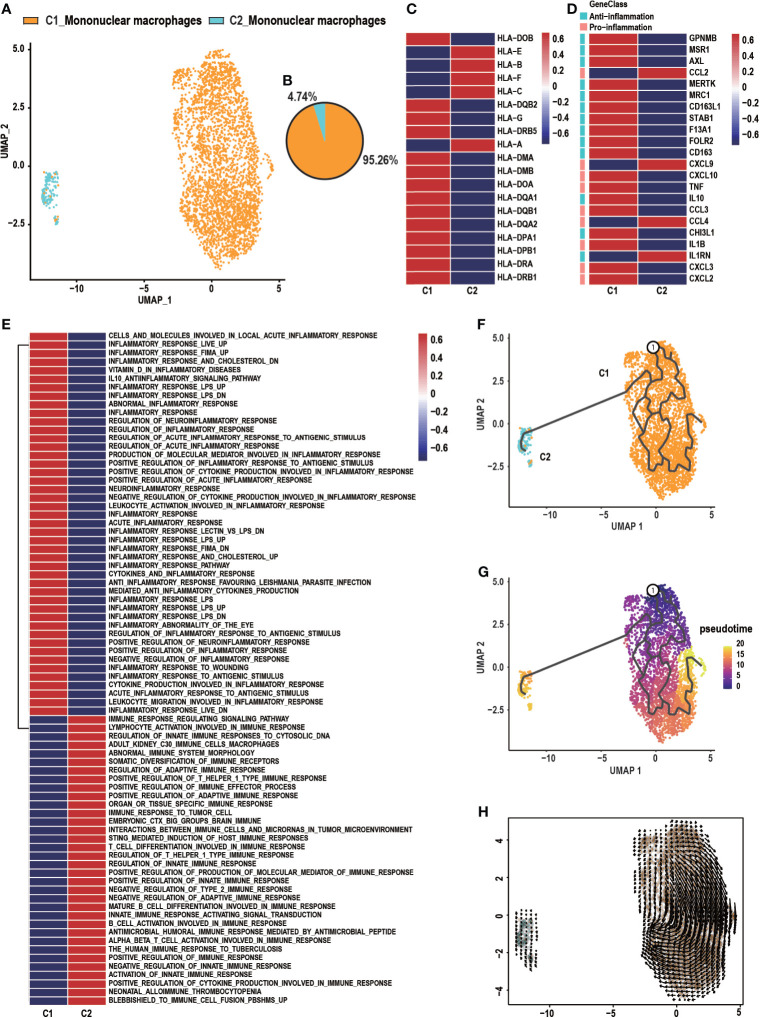
Heterogeneity of macrophage populations in GCTB. **(A)** C1_Mononuclear macrophages and C2_Mononuclear macrophages were identified by UMAP analysis. **(B)** A pie chart depicting macrophage cell composition. **(C)** Heatmap of genes involved in antigen presenting by C1_Mononuclear macrophages and C2_Mononuclear macrophages. **(D)** Heatmap of genes associated with anti- and pro-inflammation properties of C1_Mononuclear macrophages and C2_Mononuclear macrophages. **(E)** GSVA analysis shows the function of C1_Mononuclear macrophages and C2_Mononuclear macrophages. **(F, G)** The differentiation and developmental trajectory of C1_Mononuclear macrophages and C2_Mononuclear macrophages in GCTB. **(H)** Velocity field projected onto the UMAP plot of C1_Mononuclear macrophages and C2_Mononuclear macrophages. Arrows indicate the direction of the differentiation and the average velocity. GCTB, giant cell tumor of bone; UMAP, uniform manifold approximation and projection for dimension reduction.

Previous studies found that mononuclear macrophages are critical abundant components of the tumor microenvironment and have been widely implicated in tumor stimulating and suppressing activities (31, 32). Two distinct subclusters comprised of C1_Mononuclear macrophages and C2_Mononuclear macrophages were identified in the UMAP analysis (**Figure 2A**). C1_Mononuclear macrophages were 95.26% of all mononuclear macrophages in the specimen, while 4.74% were C2_Mononuclear macrophages (**Figure 2B**). C1_Mononuclear macrophages had a high expression level of HLA-related genes, indicating that they were antigen presenting (**Figure 2C**). C1_Mononuclear macrophages were correlated with inflammation due to their higher expression of anti- and pro-inflammatory genes, while C2_Mononuclear macrophages were less correlated with inflammation (**Figure 2D**). C1_Mononuclear macrophages were enriched in gene sets associated with the inflammatory response, including the inflammatory response pathway and the inflammatory response to an antigen stimulus. C1_Mononuclear macrophages may be highly involved in the GCTB inflammatory microenvironment. C2_Mononuclear macrophages were enriched in gene sets associated with an immune response, including regulation of the innate immune response and the immune response to tumor cells, suggesting that they may be immune cells that have anti-GCTB properties (**Figure 2E**). The trajectory and RNA velocity analysis of mononuclear macrophages showed that the differentiation trajectory began with C1_Mononuclear macrophages, which can differentiate into C2_Mononuclear macrophages (**Figures 2F–H**).

### Heterogeneity of Osteoclasts in GCTB

**Figure 3:**
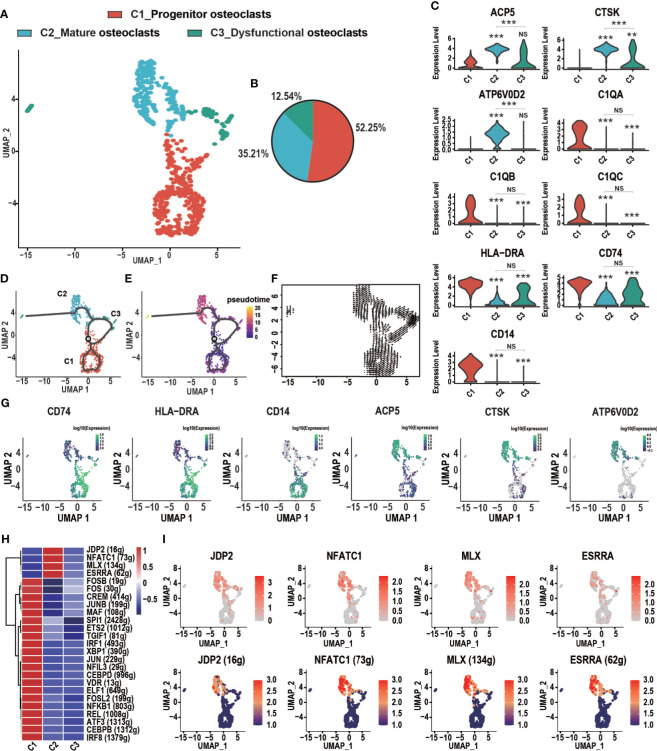
Heterogeneity of osteoclasts in GCTB lesions. **(A)** An UMAP plot shows three main subtypes of osteoclasts: C1_Progenitor osteoclasts, C2_Mature osteoclasts, and C3_Dysfunctional osteoclasts. **(B)** A pie chart depicts the cell composition of the osteoclasts. **(C)** Violin plots showing relevant marker genes in the osteoclast subtypes (**P < 0.01, ***P <0.001, NS: No statistical significance). **(D, E)** The dynamics of the osteoclast subclusters showed with a monocle 3 trajectory plot. **(F)** Velocity field projected onto the UMAP plot of the osteoclasts. Arrows indicate the direction of the differentiation and its average velocity. **(G)** The expression levels of marker genes (CD74, HLA-DRA, CD14, ACP5, CTSK, and ATP6V0D2) related to the differentiation of osteoclasts. **(H)** Heatmap of the AUC scores of transcription factor expression regulation by SCENIC. **(I)** UMAP plot of osteoclasts color-coded for expression level (up) and the AUC of the estimated regulon activity of these transcription factors (down). GCTB, giant cell tumor of bone; UMAP, uniform manifold approximation and projection for dimension reduction; SCENIC, single-cell regulatory network inference and clustering; AUC, area under the curve.

GCTB, also known as “osteoclastoma,” is thought to be highly associated with osteoclasts, which play a critical role in the osteolysis of GCTB (33). Three distinct subtypes of osteoclasts were identified according to known marker genes (**Figures 3A–C**). These subclusters were described as follows: (1) C1_Progenitor osteoclasts, which accounted for most osteoclasts (52.25%) and expressed high levels of myeloid cell markers such as C1QA, C1QB, HLA-DRA, major histocompatibility complex II (MHC-II; CD74), and CD14; (2) C2_Mature osteoclasts, which accounted for 35.21% of osteoclasts and demonstrated high levels of the osteoclast markers tartrate acid phosphatase (ACP5), cathepsin K (CTSK), and the D2 isoform of vacuolar (H^+^) ATPase H^+^ Transporting V0 Subunit D2 (ATP6V0D2), which are necessary for the maturation of osteoclasts (34, 35); and (3) C3_Dysfunctional osteoclasts, which accounted for 12.54% of all osteoclasts and poorly expressed genes related to osteoclast function. Osteoclasts followed a differentiation trajectory that mainly started from the initial cluster of C1_Progenitor osteoclasts, which differentiated into C2_Mature osteoclasts and C3_Dysfunctional osteoclasts (**Figures 3D–F**). The cell trajectory analysis of marker genes confirmed these results. CD74, HLA-DRA, and CD14 were located at the initial position of the pseudotime and the expression levels of CTSK, ACP5, and ATP6V0D2 gradually increased along the pseudotime trajectory (**Figure 3G**). The regulatory network underlying each subset of osteoclasts was examined with SCENIC, and specific TF regulons for each osteoclast subset were identified. As shown in C2_Mature osteoclasts, four genes of TFs (JDP2, NFATC1, MLX, and ESRRA) were significantly up-regulated and four TFs were activated **(** **Figures 3H, I** **)**. Jun dimerization protein 2 (JDP2) is a transcription factor of the AP-1 family that regulates osteoclast differentiation by RANKL (36, 37). The nuclear factor of activated T cells, cytoplasmic 1 (NFATc1), which belongs to the NFAT family, plays a vital role in osteoclast formation and function (38). MLX can promote myogenesis by inducing the expression of several myokines, including insulin-like growth factor 2 (IGF2) (39, 40). ESRRA is a transcription factor that is involved in tumorigenesis, such as oral squamous cell carcinoma (41).

**Figure 4:**
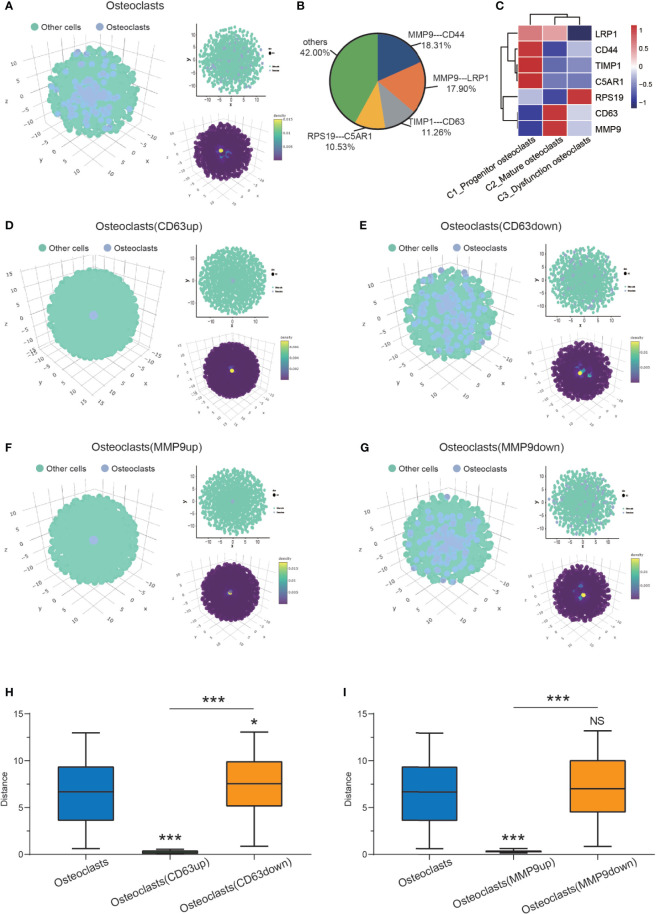
CSOmap revealing osteoclast spatial information. **(A)** Global (left, lower right) and cross-sectional (upper right) views of the spatial information of osteoclasts and other cells. **(B)** Contributions of all ligand-receptor genes to the interactions between osteoclasts. **(C)** Heatmap of the 4 pairs of ligand receptors: (MMP9-CD44, MMP9-LRP1, TIMP1-CD63, RPS19-C5AR1). **(D, E)** Global (left, lower right) and cross-sectional (upper right) views of the spatial information on osteoclasts and other cells after the overexpression and knockdown of CD63. **(F, G)** Global (left, lower right) and cross-sectional (upper right) views of the spatial information of osteoclasts and other cells after the overexpression and knockdown of MMP9. **(H)** The distance of osteoclasts (normal control/CD63up/down) to the center (*P < 0.05, ***P < 0.001). **(I)** The distance of osteoclasts (normal control/CD63up/down) to the center (***P < 0.001; NS, No statistical significance). CSOmap, Cellular Spatial Organization mapper.

Spatial information of osteoclasts and other cells (mononuclear macrophages, osteoblasts, NK/T cells, pericytes, proliferating cells, endothelial cells, and chondrocytes) was obtained with CSOmap analysis (**Figure 4A**). Four pairs of ligand receptors contributed more than 10% to the spatial reconstruction of the cell (**Figure 4B**). Based on the analysis of the expression levels of these ligand receptor pairs (MMP9-CD44, MMP9-LRP1, TIMP1-CD63, RPS19-C5AR1), it was found that CD63 and MMP9 were highly expressed in C2_mature osteoclasts (**Figure 4C**). Through computer simulation of CD63 overexpression osteoclasts were found to have a closer spatial structure, suggesting that they were in a state of aggregation at this time (**Figure 4D**). When CD63 was knocked down, osteoclasts had a looser spatial structure (**Figure 4E**), suggesting a state of dispersion. We quantified the distance between the osteoclast cells and the pseudo-space center to further confirm the visual features (**Figure 4H**). Similarly, through computer simulation the overexpression of MMP9 led to a closer osteoclast spatial structure (**Figure 4F**), while knockdown of MMP9 led to a looser spatial structure (**Figure 4G**). However, the change in the distance between osteoclast cells and the pseudo-space center after MMP9 knock-down was not statistically significant (**Figure 4I**).

### Heterogeneity of NK/T Cells

**Figure 5:**
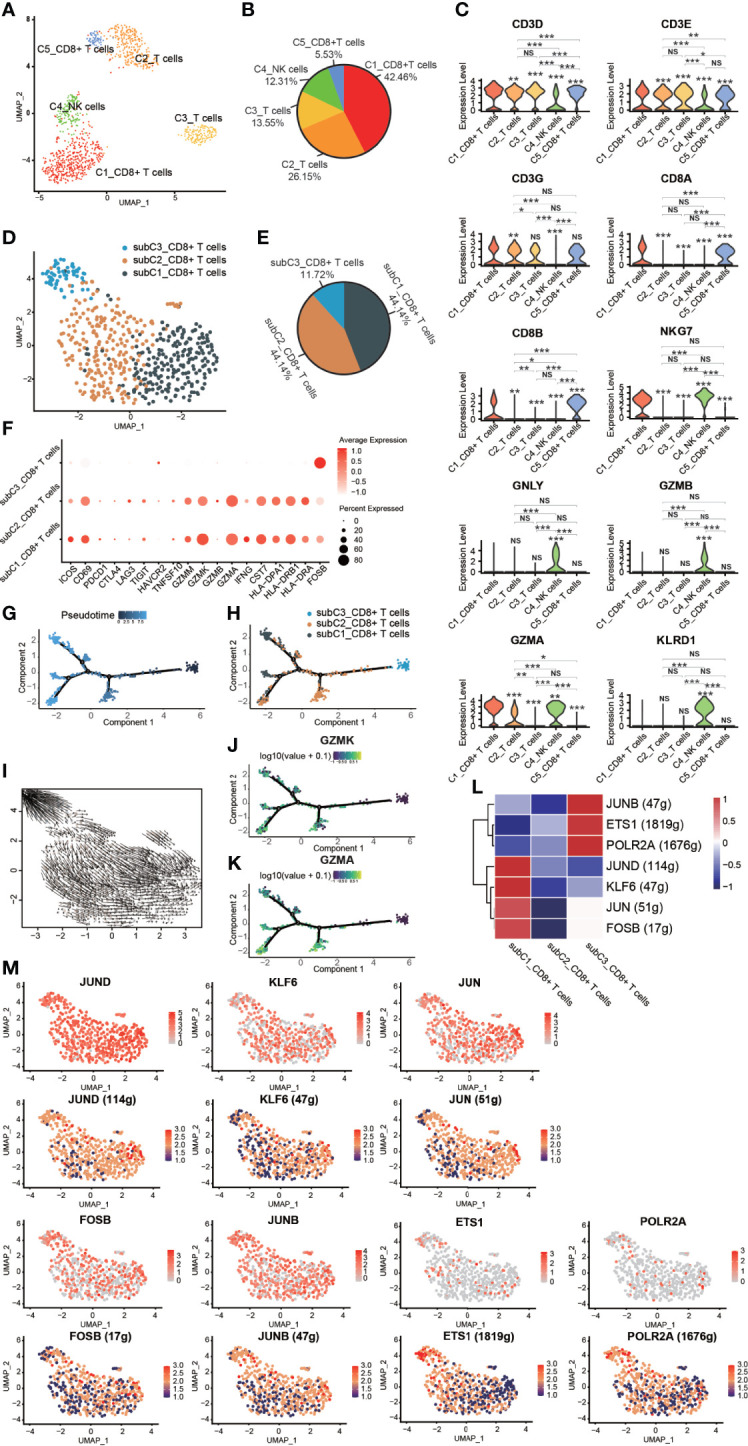
Heterogeneity of NK/T cells in GCTB. **(A)** UMAP plot showing different subtypes of NK/T cells. **(B)** A pie chart depicting the subtypes composition of NK/T cells. **(C)** Violin plots displaying the expression of representative, well-known markers across the NK/T cells types identified in GCTB (*P <0.05, **P <0.01, ***P <0.001, NS: No statistical significance). **(D)** UMAP plot showing different subtypes of CD8^+^ T cells. **(E)** A pie chart indicating the subtypes composition of CD8^+^ T cells. **(F)** Dot-plot of marker genes in subtypes of CD8^+^ T cells. Shades of red represent the expression level, and the dot sizes represent the relative abundance of each gene. **(G, H)** The dynamics of CD8^+^ T cell subtype as shown by the monocle 2 trajectory plot. **(I)** An RNA velocity field projected onto the UMAP plot of the subtype of CD8^+^ T cells. Arrows indicate the direction of the differentiation and its average velocity. **(J, K)** The expression levels of the marker genes GZMK and GZMA, which are related to the differentiation of a subtype of CD8^+^ T cells. **(L)** Heatmap of the AUC scores of expression regulation by transcription factors as estimated by SCENIC. **(M)** UMAP plot of CD8^+^ T cell subtype, the expression level (up) and for the AUC of the estimated regulon activity of these transcription factors (down). GCTB, giant cell tumor of bone; UMAP, uniform manifold approximation and projection for dimension reduction; NK cells, natural killer cells; GZMK, Granzyme K; GZMA, Granzyme A.

NK/T cells play an essential role in mediating response to chemotherapy and improving clinical outcomes in various cancers, including liver cancer (42), ovarian cancer (43), and breast cancer (44). Five subclusters of cells were identified from the GCTB lesion: C1_CD8^+^ T cells, C2_T cells, C3_T cells, C4_NK cells and C5_ CD8^+^ T cells (**Figures 5A, B**). C1_CD8^+^ T cells, and C2_CD8^+^ T cells shared specific T-cell markers genes, such as CD3D, CD3E, CD3G, CD8A, and CD8B (19, 45). C2_T cells and C3_T cells expressed CD3D, CD3E, and CD3G. C4_NK cells significantly expressed NKG7, GNLY, GZMA, GZMB, and KLRD1, which are thought to be the canonical marker genes of NK cells (23, 46) (**Figure 5C**). Natural killer cell granule protein 7 (NKG7) is a marker of NK cells that is critical for controlling cancer initiation, growth, and metastasis. NKG7 was highly expressed in C1_CD8^+^ T cells, suggesting the abundant infiltration of NK/T cells in GCTB.

Three distinct subtypes of CD8^+^ T cells were identified according to known marker genes (**Figures 5D–F**). These subclusters were as follows: (1) subC1_CD8^+^ T cells accounted for 44.14% of all CD8^+^ T cells and expressed low levels of markers related to T cell function such as PDCD1, CTLA4, LAG3, TIGIT, and HAVCR2. However, subC1_CD8^+^ T cells had a relatively high expression levels of CD69, GZMM, GZMK, GZMA, CST7, HLA-DPA1, HLA-DRB1 and HLA-DRA; (2) subC2_CD8^+^ T cells, which accounted for 44.14% of all CD8+ T cells and expressed the same markers as subC1_CD8^+^ T cells, but at lower levels; and (3) subC3_CD8^+^ T cells, which accounted for 11.72% of all CD8+ T cells and expressed high levels of FOSB, a marker gene of early-stage CD8^+^ T cells. CD8^+^ T cells followed a differentiation trajectory that mainly started from the initial cluster of partial subC3_CD8^+^ T cells, which differentiated into subC2_CD8^+^ T cells and subC1_CD8^+^ T cells (**Figures 5G–I**). The expression levels of GZMK and GZMA gradually increased along the pseudotime trajectory (**Figurse 5J, K**). Four TFs (FOSB, JUN, KLF6, and JUND) were slightly up-regulated and 4 TFs were activated in subC1_CD8^+^ T cells, while three TFs (POLR2A, ETS1 and JUNB) were slightly up-regulated and 3 TFs were activated in subC3_CD8^+^ T cells **(** **Figures 5L, M** **)**.

FOSB, a member of the Fos transcription factor family and a component of the AP-1 complex, has been implicated in diverse biological processes including bone-forming tumors and a subset of vascular tumors (47–49). JUN, an extensively studied protein of the AP-1 complex, is involved in numerous cell activities such as proliferation, apoptosis, survival, tumorigenesis, and tissue morphogenesis (50). Kruppel like factor 6 (KLF6), a transcription factor of the zinc finger family, has been reported to play an important role in lipid homeostasis regulation in clear cell renal cell carcinoma (51). Jund proto-oncogene subunit (JunD), a prominent AP-1 component, is a bone formation inhibitor that contributes to low bone mass induced by estrogen depletion (52). RNA polymerase II subunit A (POLR2A), one of the subunits of the human RNA polymerase II complex, encodes the largest subunit that is indispensable for polymerase activity during messenger RNA synthesis. Prior work suggested that inhibiting POLR2A with a-amanitin could lead to extensive cell death (53). ETS1 belongs to a large family of the ETS domain family of transcription factors and is thought to be linked to poor survival during cancer progression (54) as well as transcription networks associated with CD8 T cell differentiation (55). JUNB, an AP-1 transcription factor, is expressed by eTreg cells and promotes an IRF4-dependent transcription program (56).

### Complex Intercellular and Molecular Interaction Networks in GCTB

**Figure 6:**
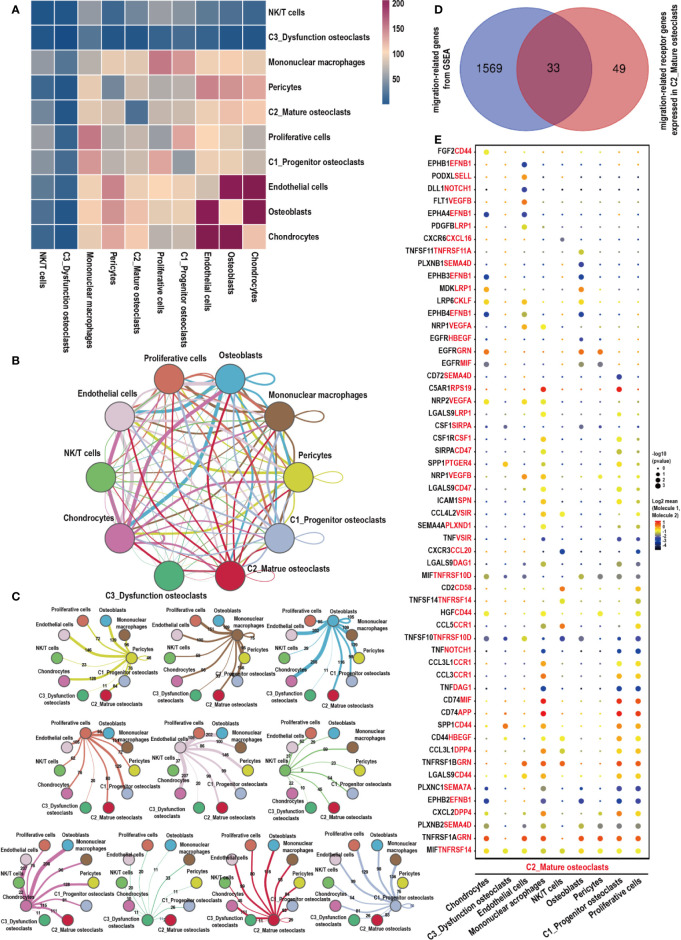
Cell–cell communication network of CellPhoneDB in the GCTB. **(A)** Heatmap showing the number of potential ligand–receptor pairs among the predicted cell types. **(B, C)** Interaction network constructed by CellPhoneDB. Thicker lines indicate more interaction with other types of cells. **(D)** Venn diagram of genes related to cells migration obtained from GSEA, and those that acted as receptors expressed in C2_Mature osteoclasts in our data. **(E)** Ligand–receptor pairs detected with CellPhoneDB are shown in a bubble plot. GCTB, giant cell tumor of bone; UMAP, uniform manifold approximation and projection for dimension reduction; GSEA, gene set enrichment analysis.

CellPhoneDB analysis was used to calculate the number of ligand–receptor pairs among all of the cell types in our data (**Figure 6A**). These ligand-receptor relationships are shown in **Figures 6B, C**. Thirty-three migration-associated genes were expressed by C2_Mature osteoclasts (**Figure 6D**, **Supplementary Table 2**). Cells of interest were selected, and their ligand–receptor relationships were identified (**Figure 6E**). All these ligand–receptor pairs were expressed individually. All cells expressed the ligand MIF, which is a macrophage migration inhibitory factor that regulates the function of mononuclear macrophages in host defense at sites of inflammation, while its receptor TNFRSF14 was expressed in C2_Mature osteoclasts. Osteoblasts significantly expressed TNFSF11 (RANKL), while the corresponding receptor TNFRSF11A (RANK) was expressed by C2_Mature osteoclasts. The TNFSF11/TNFRSF11A pathway regulates the activation of osteoclasts and induces the migration of tumor cells, notably in breast and lung cancer (57). CKLF (chemokine-like factor) is a novel cloned chemotactic cytokine that regulates the migration of immune cells (58) and was significantly expressed by C2_Mature osteoclasts while its receptor LPR6 was expressed in chondrocytes, endothelial cells, and osteoblasts. We also measured the expression levels of migration-related genes in the different cells (**Supplementary Figure 2A**, **Supplementary Table 3**). ACP5, CTSK, and ATP6V0D2, which are associated with the function of osteoclasts, were highly expressed in C2_Mature osteoclasts. Further, genes associated with cell migration were mostly expressed by C1_Progenitor osteoclasts (for instance, PTGER4, HBEGF, VEGFA, CD44, and VSIR) and C2_Mature osteoclasts (such as TNFRSF14, CD58, DPP4, EFNB1, SEMA7A, GRN, TNFRSF11A, APP, and CKLF). CKLF was more highly expressed by C2_Mature osteoclasts than by C1_Progenitor osteoclasts and C2_Mononuclear macrophages. Multiplex immunofluorescence experiments also revealed the presence of C2_Mature osteoclasts (**Supplementary Figure 2B**).

**Figure 7:**
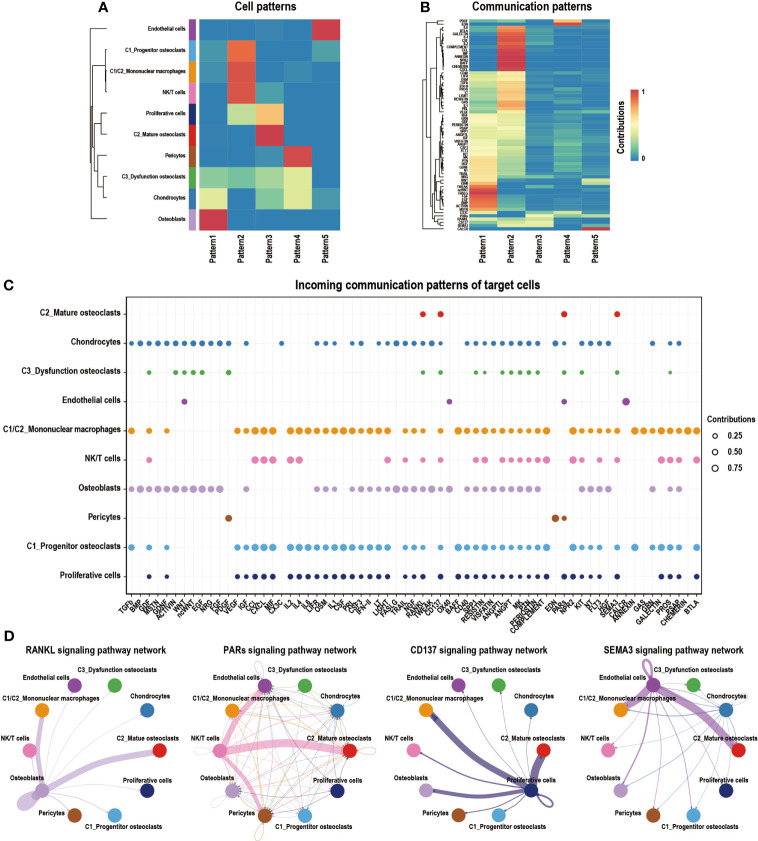
Cell–cell communication network of CellChat in GCTB. **(A)** Heatmap showing the enrichment of different cell types in different modules. The deeper red the color is, the higher its degree of enrichment. **(B)** Heatmap showing the contribution of different signaling pathways in different expression modules. The deeper the red color is, the higher the contribution of the signaling pathways. **(C)** A bubble plot showing the signaling pathways of interest in GCTB as detected by CellChat. **(D)** An interaction network shows the ligand–receptor pairs that have a known biological significance. GCTB, giant cell tumor of bone.

We algorithmically divided all of the cells into five patterns *via* CellChat analysis. C2_Mature osteoclasts mainly gathered in Pattern3, which expressed the RANKL, PARs, CD137 RANKL, and SEMA3 signaling pathways (**Figures 7A, B**). The bubble diagram shows the expression of these signaling pathways by C2_Mature osteoclasts (**Figure 7C**). Signaling pathways of interest were then selected, and their interaction networks are shown (**Figure 7D**). The RANKL signaling pathway was found to be highly associated with osteoclast formation and migration (57). Proteinase-activated receptors (PARs), which are key components of the PARs signaling pathway, are expressed in various epithelial tumors and can regulate progression and metastasis (59). The CD137 signaling pathway is critically involved in the promotion of breast cancer bone metastasis *via* enhancement of the migration and differentiation of monocytes/macrophages into osteoclast (60). SEMA3, a subfamily of signaling molecules in the SEMA3 signaling pathway, plays diverse roles in the regulation of cell proliferation, differentiation, and migration (61).

**Figure 8:**
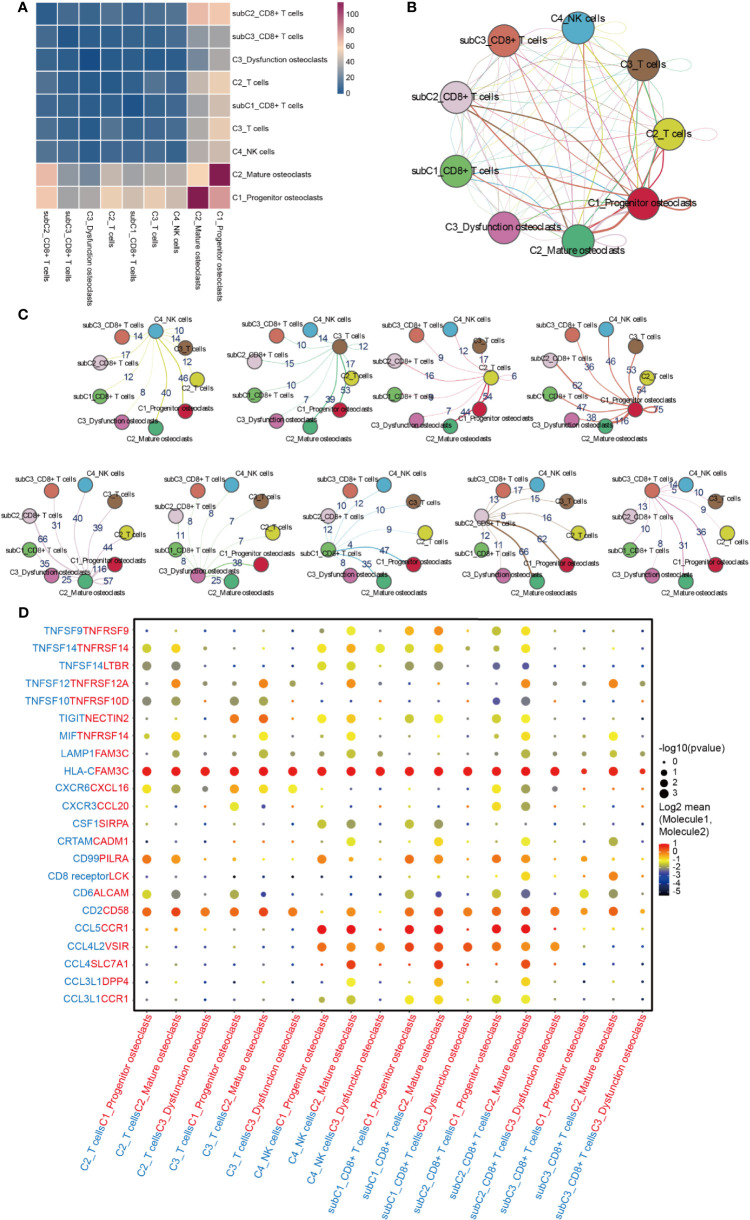
Cell-cell communication network between NK/T cells and osteoclasts in GCTB. **(A)** Heatmap showing the number of potential ligand-receptor pairs among the selected cell types. **(B, C)** Interaction network constructed by CellPhoneDB. Thicker lines mean more interaction with other types of cells. **(D)** Ligand-receptor pairs are shown in the bubble plot. GCTB, giant cell tumor of bone.

The interaction between NK/T cells and osteoclasts was analyzed (**Figures 8A–C**). Cells of interest were selected, and their ligand-receptor interactions were identified (**Figure 8D**). All immune cells expressed ligand HLA-C, a type of human leucocyte antigen that is correlated with the enhanced potent cytotoxic response of T cells immunity (62). Its receptor FAM3C, a member of the FAM3C gene family that is related to the inflammatory response and bone remodeling (63), was expressed in all subtypes of osteoclasts. C2_Mature osteoclasts expressed higher levels of TNFRSF14, while its receptor MIF, which is macrophage migration inhibitory factor that regulates the function of mononuclear macrophages in host defense at sites of inflammation, was expressed in C2_T cells, C3_T cells, C4_NK cells, subC2_CD8^+^T cells, and subC3_CD8^+^T cells. In addition to HLA-C_FAM3C, CD2_CD58 was also more highly expressed by these immune cells than osteoclasts. CXCR6 was significantly expressed by C2_T cells, C3_T cells and subC2_CD8^+^ T cells, while its receptor CXCL16 was expressed in all sub types of osteoclasts. CXCR6 has been shown to draw CD8^+^ T cells to the liver in graft-versus-host disease and is required for the maintenance of liver-resident CD8^+^ T cells following infection (64, 65). CXCL16 is a chemokine that binds to CXCR6 on Th1 and activated CD8 effector T cells and plays an important role in their recruitment to sites of inflammation (66, 67).

## Discussion

The existing literature on GCTB is mostly comprised of genetic and genomic studies and clinical case reports (68, 69). These studies provide a basis for understanding the current treatment strategies for GCTB. However, accurate analysis of the cellular heterogeneity of GCTB remains challenging. To address the heterogeneity of GCTB, the present study evaluated a patient who was diagnosed with GCTB. To the best of our knowledge, this is the first comprehensive single-cell transcriptome study on a patient with GCTB. Khazaei et al. (70) characterized the transcriptomic of G34W GCTB and reported that a G34W mutation is necessary for GCTB tumorigenesis. However, the number of GCTB subtypes, their distinctive properties, and their heterogeneity remains unclear. In the present report, we identified eight major cell clusters in GCTB with UMAP clustering. The complexity of the GCTB cellular ecosystem and the intratumor heterogeneity of GCTB were revealed by scRNA-seq.

The main clinical manifestation of GCTB is osteolysis, which can lead to significant motor dysfunction. Osteoclasts, the only cells that are known to be involved in osteolysis in the human body, are believed to be intensively involved with GCTB (71). An increasingly large body of evidence has shown that key genes such as ACP5, CTSK, and ATP6V0D2 (72) are involved in the bone resorption function of osteoclasts. The directional migration of osteoclasts at the lesion site also attracted our interest. C1_Progenitor osteoclasts and C2_Mature osteoclasts have their own unique genes that regulate their migration and their highly migratory state. Interestingly, our data showed the CKLF, a newly cloned chemotactic cytokine, was relatively increased in subtypes of C2_Mature osteoclasts, indicating improved osteoclast mobility during maturation. In addition, the key genes that affected the aggregation of osteoclasts in GCTB were identified and may be key targets of future therapies, although more experiments are needed to further confirm this. As the high migration state of osteoclasts was confirmed, analysis of key ligand–receptor pairs that regulate the interactions of the cells within the tumor environment of GCTB is important. Osteoclasts were regulated by several cells in GCTB. We mainly focused on the key ligand-receptor pairs that interacted with and regulated C2_Mature osteoclasts and other cells in the GCTB tumor microenvironment. Previous reports showed that TNFRSF11A_TNFSF11, a classical ligand-receptor pair that regulates osteoclast maturation and function (73), was also included in our data. These key ligand–receptor pairs provide strong evidence of molecular crosstalk in the bone microenvironment (74) and can serve as guidance for follow-up studies on osteoclast migration and function. Moreover, four key signaling pathways involved in the formation and migration of C2_Mature osteoclasts were identified: the RANKL signaling pathway network, the CD137 signaling pathway network, the PARs signaling pathway network, and the SMEA3 signaling pathway network. The results provide a theoretical basis for future studies on the osteolytic effects of GCTB.

There are several limitations of this study: 1. scRNA-seq only contains about 0.1 pg of mRNA per cell on average, so there are too few materials available for sequencing (75); 2. the single-cell suspension digestion scheme directly affects the composition ratio of cells, resulting in the loss of rare cells, increasing cell stress and affecting cell gene expression (9); 3. restrictions on the depth of the sequencing, as at present, most scRNA-seq can only detect 10%-20% of mRNA (76, 77); 4. the small number of GCTB cases used in this work can only reflect the heterogeneity of the cells rather than between patients, and the results may be biased due to the small number of included cases.

Overall, this study characterized the heterogeneity of GCTB, provided a valuable single-cell transcription atlas and novel insights into GCTB, and identified a new mechanism and target for clinical GCTB treatment.

## Data Availability Statement

The datasets presented in this study can be found in online repositories. The names of the repository/repositories and accession number(s) can be found below: https://www.ncbi.nlm.nih.gov/, GSE168664.

## Ethics Statement

The studies involving human participants were reviewed and approved by The First Affiliated Hospital of Guangxi Medical University (No.2019KY-E-153). The patients/participants provided their written informed consent to participate in this study.

## Author Contributions

QW, YL, and JM were responsible for the conception of the study. WF, ZQ, QH, and MH harvested the GCTB tissue and performed the scRNA-seq experiments. WF, MH, XJ, and CL analyzed the data. MH, TX, HL, and SL performed the immunofluorescence staining. WF, MH, and JH wrote the paper. QW, YL, JM, and JX contributed to the revision and discussion of the paper. All authors contributed to the article and approved the submitted version.

## Funding

This project was supported in part by the Guangxi Key Laboratory of Genomics and Personalized Medicine. This project was also supported by the Nanning Scientific Research and Technology Development Plan (grant no. 20183012), Guangxi Key R&D Program (grant no.AB17292073), the National Natural Science Foundation of China (grant no. 81960768), Guangxi Key Laboratory of Genomics and Personalized Medicine open project (grant no. GXGPMC201903), Natural Science Foundation of Guangxi Province (grant no. 2020GXNSFAA259088), Guangxi Natural Science Foundation of Youth Science Foundation project (grant no. 2017GXNSFBA198098), the “Medical Excellence Award” Funded by the Creative Research Development Grant from the First Affiliated Hospital of Guangxi Medical University, and the Youth Science and Technology Project of the First Affiliated Hospital of Guangxi Medical University (grant no. 201903038). YL and SL were visiting scholars to the Molecule Biology Lab at the University of Western Australia.

## Conflict of Interest

The authors declare that the research was conducted in the absence of any commercial or financial relationships that could be construed as a potential conflict of interest.

## Publisher’s Note

All claims expressed in this article are solely those of the authors and do not necessarily represent those of their affiliated organizations, or those of the publisher, the editors and the reviewers. Any product that may be evaluated in this article, or claim that may be made by its manufacturer, is not guaranteed or endorsed by the publisher.

## Supplementary Material

Supplementary Figure 1 **(A)** 99mTc-MDP Bone Scan shows a clumpy reflexive-concentrated focus in the lower right distal femur. **(B, C)** Plain radiographs shows an osteolytic lesion in the distal femur. **(D)** A sagittal T1-weighted image shows the tumor with heterogeneous signal intensity, and **(E)** cystic changes are visible on a fat-suppressed T2-weighted image. **(F)** The pathologic images show local tumor cell infiltration (100×). GCTB, giant cell tumor of bone.

Supplementary Figure 2 **(A)** Expression levels of migration-related genes between the different cells (*P < 0.05, **P < 0.01, ***P < 0.001; NS, No statistical significance). **(B)** Multiplex IHC staining of GCTB tissue. Arrows represent the co-expression positions of the relevant indicators (DAPI, ACP5, ATP6V0D2, CKLF) in Multiplex IHC (scale bar=200 μm). GCTB, giant cell tumor of bone; IHC, immunohistochemistry

Supplementary Table 1 Characteristics of the patient with GCTB included in this study.

Supplementary Table 2 Migration-associated genes.

Supplementary Table 3 Expression levels of migration-related genes.
