# Supplementary figures and images for: Single-Cell RNA Sequencing Reveals the Migration of Osteoclasts in Giant Cell Tumor of Bone
Source: Front Oncol. 2021 Aug 24;11:715552. doi: 10.3389/fonc.2021.715552 (PMC8421549; doi:10.3389/fonc.2021.715552)

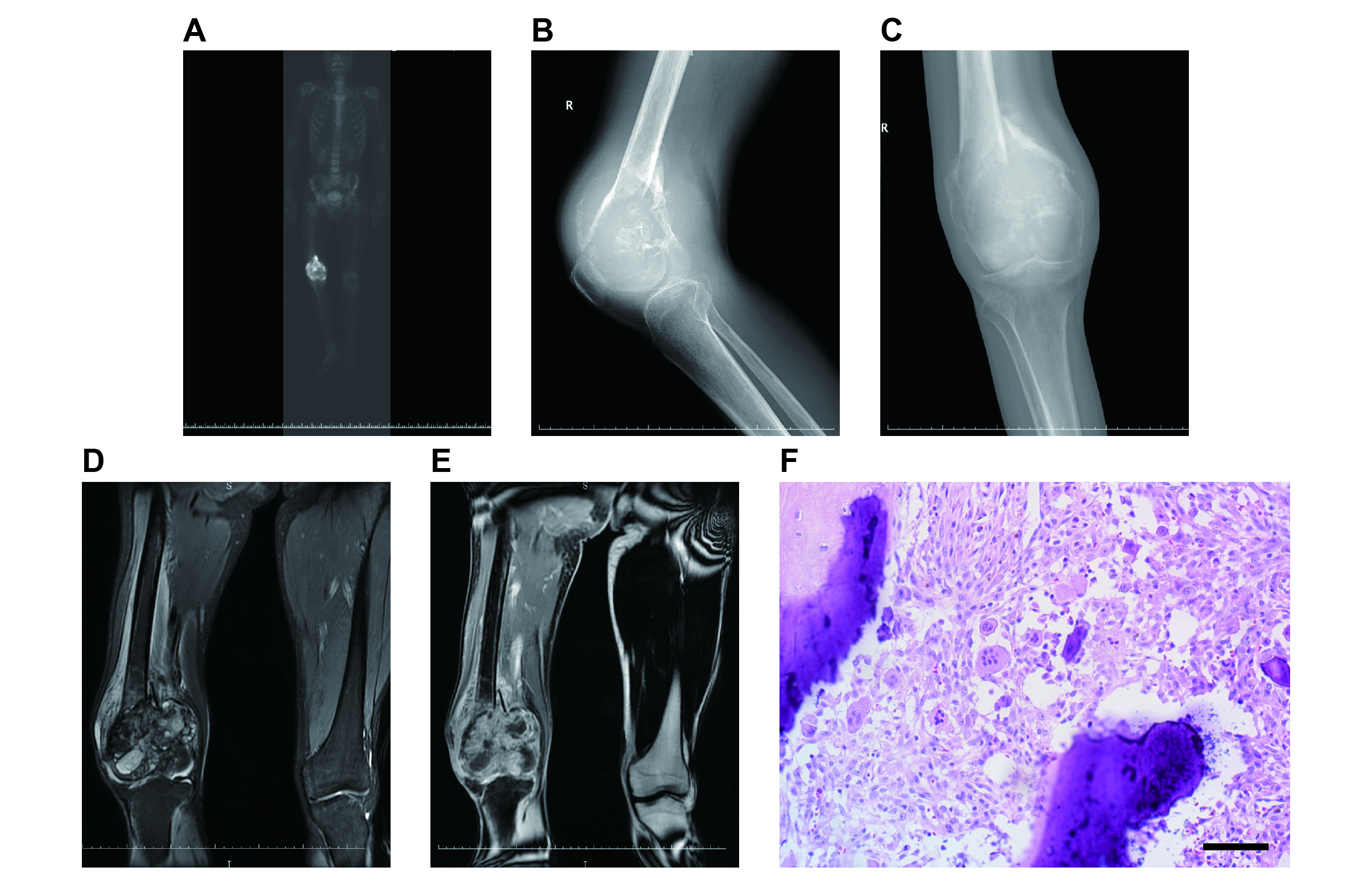

Supplement: Supplementary Figure 1 — (A) 99mTc-MDP Bone Scan shows a clumpy reflexive-concentrated focus in the lower right distal femur. (B, C) Plain radiographs shows an osteolytic lesion in the distal femur. (D) A sagittal T1-weighted image shows the tumor with heterogeneous signal intensity, and (E) cystic changes are visible on a fat-suppressed T2-weighted image. (F) The pathologic images show local tumor cell infiltration (100×). GCTB, giant cell tumor of bone. [file Image_1.jpeg]

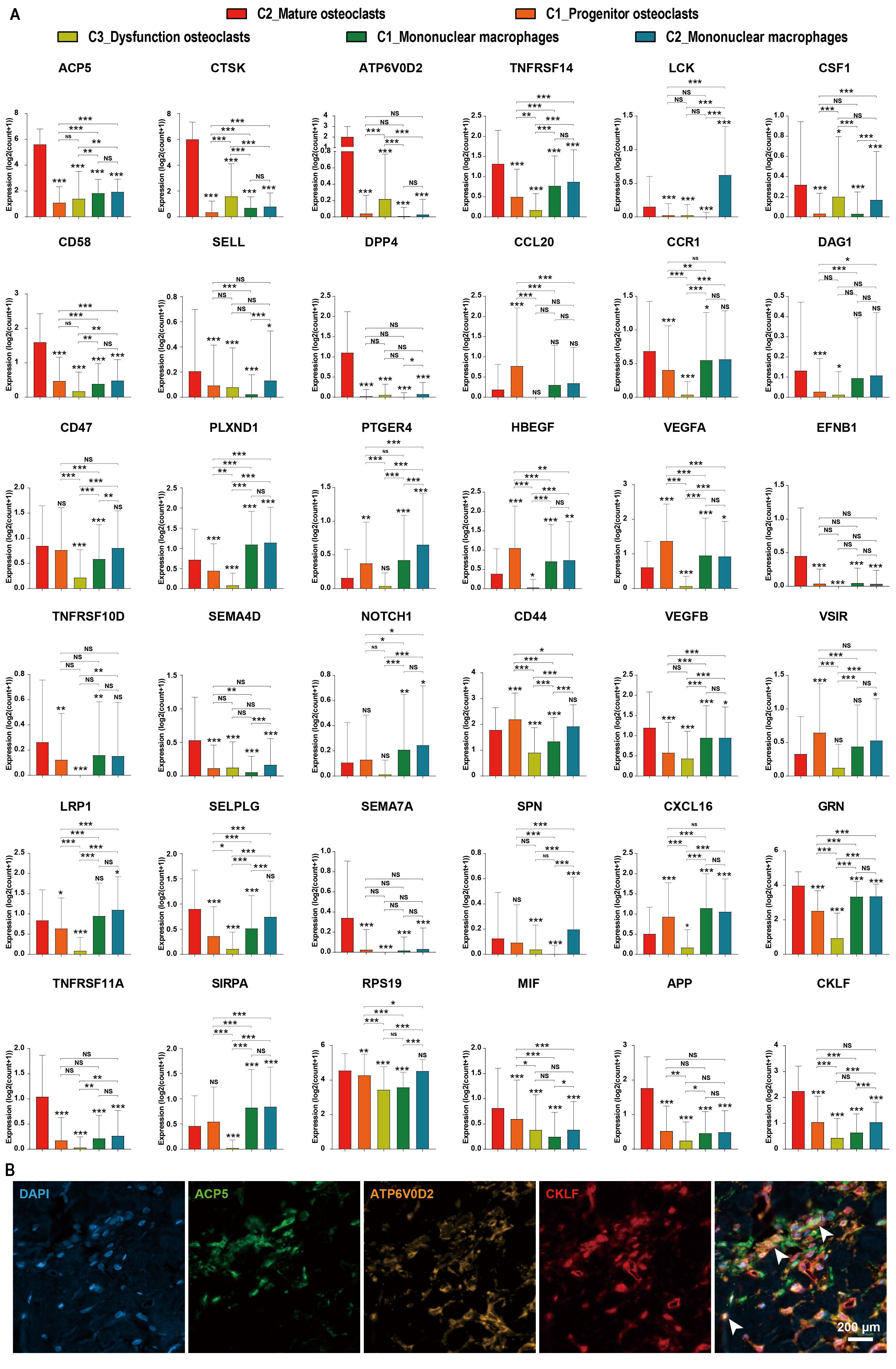

Supplement: Supplementary Figure 2 — (A) Expression levels of migration-related genes between the different cells (*P < 0.05, **P < 0.01, ***P < 0.001; NS, No statistical significance). (B) Multiplex IHC staining of GCTB tissue. Arrows represent the co-expression positions of the relevant indicators (DAPI, ACP5, ATP6V0D2, CKLF) in Multiplex IHC (scale bar=200 μm). GCTB, giant cell tumor of bone; IHC, immunohistochemistry [file Image_2.jpg]
