# Supplementary material for: Single-Cell RNA Sequencing Reveals the Migration of Osteoclasts in Giant Cell Tumor of Bone
Source: Front Oncol. 2021 Aug 24;11:715552. doi: 10.3389/fonc.2021.715552 (PMC8421549; doi:10.3389/fonc.2021.715552)
Supplement: Supplementary Table 1 — Characteristics of the patient with GCTB included in this study. [file Table_1.docx]

| Patient ID | Sex | Age | Location | Size | Tissue samples | Histological diagnosis |
| --- | --- | --- | --- | --- | --- | --- |
| GCTB-001 | Male | 39 | Distal femur | 9cm×7cm×5cm | Tumor tissue | Giant cell tumor of bone |

Supplementary Table 1. Characteristics of the patient with GCTB included in this study
